# Public Talks and Science Listens: A Community-Based Participatory Approach to Characterizing Environmental Health Risk Perceptions and Assessing Recovery Needs in the Wake of Hurricanes Katrina and Rita

**DOI:** 10.4137/EHI.S2158

**Published:** 2009-06-23

**Authors:** J. Sullivan, B. Parras, R. St. Marie, W. Subra, S. Petronella, J. Gorenstein, R. Fuchs-Young, R.K. Santa, A. Chavarria, J. Ward, P. Diamond

**Affiliations:** 1 University of Texas Medical Branch @ Galveston/NIEHS Center in Environmental Toxicology; 2 University of Texas/MD Anderson Cancer Center @ Smithville/Center for Research in Environmental Diseases; 3 T.e.j.a.s. (Texas Environmental Justice Advocacy Services (Houston TX); 4 Inner-Works, Inc (Larose LA); 5 Subra Company (New Iberia LA). Email: josulliv@utmb.edu

**Keywords:** risk perception, risk communication, local knowledge, exposure pathway, project CEHRO, disaster management, CBPR, environmental justice, NIEHS

## Abstract

In response to the human health threats stemming from Hurricanes Katrina and Rita, inter-disciplinary working groups representing P30-funded Centers of the National Institute Environmental Health Sciences were created to assess threats posed by mold, harmful alga blooms, chemical toxicants, and various infectious agents at selected sites throughout the hurricane impact zone. Because of proximity to impacted areas, UTMB NIEHS Center in Environmental Toxicology was charged with coordinating direct community outreach efforts, primarily in south Louisiana. In early October 2005, UTMB/NIEHS Center Community Outreach and Education Core, in collaboration with outreach counterparts at The University of Texas MD Anderson Cancer Center @ Smithville TX/Center for Research in Environmental Disease sent two groups into southern Louisiana. One group used Lafourche Parish as a base to deliver humanitarian aid and assess local needs for additional supplies during local recovery/reclamation. A second group, ranging through New Iberia, New Orleans, Chalmette, rural Terrebonne, Lafourche and Jefferson Parishes and Baton Rouge met with community environmental leaders, emergency personnel and local citizens to 1) sample public risk perceptions, 2) evaluate the scope and reach of ongoing risk communication efforts, and 3) determine how the NIEHS could best collaborate with local groups in environmental health research and local capacity building efforts. This scoping survey identified specific information gaps limiting efficacy of risk communication, produced a community “wish list” of potential collaborative research projects. The project provided useful heuristics for disaster response and management planning and a platform for future collaborative efforts in environmental health assessment and risk communication with local advocacy groups in south Terrebonne-Lafourche parishes.

## Introduction

**Figure 1.**
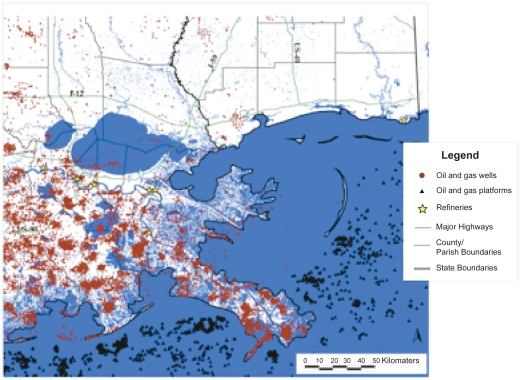
Distribution of Petro. Exploration/Production Sites: Maps of refineries, oil/gas wells, gas stations, petroleum storage stations, extraction sites.^1^

**Figure 2.**
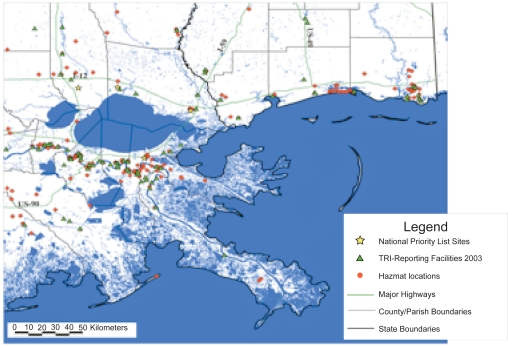
Potential Exposure Hazards: Map of National Priority List (NPL Superfund) sites, TRI reporting facilities, surface water intakes and HAZMAT sites.^1^

When Hurricane Katrina—a strong category 3 tropical cyclone with sustained 125 mph winds and a storm surge range of 12–15 feet—made landfall near Buras-Triumph LA in southern Plaquemines Parish, the environmental community was acutely aware of the potential for catastrophic damage to the regional ecosystem and the huge negative implications for public health. Impacted areas in coastal Louisiana and Mississippi “hosted” no fewer than 65 National Priority List (Superfund), Toxic Release Inventory (TRI) reporting and hazardous materials (HAZMAT) storage sites, and the bayous are literally festooned with hundreds of natural gas and petroleum exploration, production and transmission operations. The high density of offshore drilling rigs and active wells closely proximate to the Louisiana and Mississippi coasts compounded the risk of widespread destruction to community water supplies and the regional fishery from massive oil spills.^1^ (See Fig. 1 and 2).

Hurricane Rita’s impact on the coasts of east Texas and southwestern Louisiana three weeks later, amplified these concerns and galvanized action from the scientific community. In mid-September 2005, a consortium of National Institute Environmental Health Sciences (NIEHS) centers nation-wide formed to focus on human health threats stemming from the impacts of Hurricanes Katrina and Rita. These groups assessed environmental threats posed by mold, water-borne pathogens, harmful algal blooms, chemical toxicants, and various infectious agents at selected sites throughout the hurricane impact zone. Because of its proximity to the impacted areas, the University of Texas Medical Branch NIEHS Center in Environmental Toxicology was charged with coordinating direct community outreach efforts in Louisiana and East Texas.

In early October, UTMB/NIEHS Center Community Outreach and Education Core and counterparts at The University of Texas MD Anderson Cancer Center at Smithville TX/Center for Research in Environmental Disease sent two groups into Southern Louisiana. One group, based in Larose LA (Lafourche Parish) delivered humanitarian aid and assessed local needs for additional supplies during the recovery/reclamation stages of the local disaster response. A second group, ranging through New Iberia, New Orleans, Chalmette, rural Terrebonne, Lafourche and Jefferson Parishes, and Baton Rouge sampled public risk perceptions, evaluated the scope and reach of existing risk communication efforts, and attempted to determine how NIEHS could best collaborate in environmental health research and local capacity building efforts. Using open-ended interview questions, outreach personnel identified information gaps and areas of unresolved concern which limited the efficacy of risk communication, produced a community “wish list” of collaborative research, and developed a base for future efforts in environmental health and risk communication. Survey responses were compiled and presented at the NIEHS Center Directors meeting (Nov. 2005; Vanderbilt University, Nashville TN). Results informed the design, content and structure of Project CEHRO (Community Environmental Health and Risk Outreach), a follow-up intervention based on a site-specific coastal parish model addressing disaster preparedness, and environmental health and safety risks from future storms.

## Methodology

The NIEHS COEC scoping project assessed 1) perceptions of physical and social damage, 2) perceptions of environmental risk, 3) efficacy of risk communication efforts, 4) scope of immediate to long-term recovery needs, 5) range of suggestions for collaborative environmental health research. The interviews consisted of five items organized around the following central principle: *How may environmental health research institutions collaborate with local/regional stakeholders to comprehensively address environmental threats from Hurricanes Katrina, Rita and future storms?*

The method underpinning this research derives from the rubric of community-based participatory technique; Rutgers University political scientist, Frank Fischer, describes this practice as “a collaborative orientation that requires an inquiry process which informs the goals and purposes of the research, and the design of necessary interventions”.^2^ Interviews were structured for bi-directional flow of information including researchers and respondents in an active, Socratic exploration of concepts and site-specific details of risk.^3^ Since risk characterization is only as effective as the scope and range of possible inputs, tapping local knowledge and perceptions theoretically extends the data-base and widens representation among affected stakeholders.^4^,^5^

This strategy—informed by CBPR models of population health research—inverts the normal paradigm of experts assessing risk and communicating prescriptions to affected populations.^6^,^7^ By encouraging citizens from storm impact zones to assess physical and social damage, prioritize needs and identify salient environmental threats to health, we hoped to inform and expand “expert-driven” recovery, health and preparedness measures with knowledge rooted in local cultures and geography and to develop collaborative structures for health effects research, environmental monitoring, outreach and education.^8^–^10^

Core questions were informed by environmental justice/social epidemiology concepts central to community health practice: race, political marginalization, economic/social opportunities, types of work and work hazards, access to quality health care, cumulative burdens of stress, specific community vulnerabilities, and ability to recover from adverse stressors.^9^–^11^,^13^–^16^

### Design of interviews

Our interview instrument consisted of four general queries, and a fifth question reserved for directors or active members of public or environmental health organizations in Louisiana. Item #4 was designed to elicit specific information on possible foci and structures for environmental health collaborations. Items #1, #2 and #3 covered the general domains of damage/risk assessment, risk perception, reactions to official attempts at risk communication, and comments on social outcomes. (see Table 1)

### Respondent selection

Many respondents (9/15) in our purposive sample were selected on the basis of involvement in hurricane relief efforts, environmental credibility and/or direct prior connection with NIEHS Public Forum and Toxics Assistance division programs or presentations. 27% of respondents (4/15) were considered significant leaders in the Louisiana environmental community with national profiles, direct grass-roots connections and regional credibility. One respondent has achieved national prominence as an expert in levee design and construction, and the management of levee districts in flood prone areas. Difficulties establishing phone connections or physically finding identified respondents who evacuated high impact areas limited the scope of our contact base. Respondents represented 7 parishes, and the following communities: Baton Rouge (three respondents), Chalmette/Donaldsonville (one), New Orleans (two), New Iberia (one), Mathews (four), Dos Gris (one), Larose (one), Grand Boise (one), Golden Meadow (one). Additional information was gathered informally from residents of Galliano, Isle de Jean Charles, Port Fourchon, Grande Isle, Leeville, Chauvin, Dulac, Pointe-aux-Chenes and Montegut.

## Adaptations and Limitations

Several features of our methodology-design and the circumstances under which we deployed our interviews affect the nature of our results:

- Time limitations, disaster logistics and the need for swift response restricted scope and universality of the study. Sporadic phone and internet service made contact more difficult; extensive damage to infrastructure complicated physical access in some cases. It was often difficult for potential respondents to find time for the interview (50 minutes, on average) during day-to-day recovery operations.
- We chose a small purposive sample (N = 15) for our qualitative interview process, adapting our need for accurate information to the circumstances of disaster recovery. While our sample size (N = 15) would be considered woefully inadequate in a traditional probabilistic survey focused on statistical power and significant confidence levels, small samples are often sufficient in providing accurate qualitative information within a particular context (or domain of knowledge/experience), provided respondents “possess a certain degree of expertise about the domain of inquiry.” Guest, Bunce and Johnson posit that 4–12 interviews are sufficient to achieve agreement on basic facts and a representative spectrum of perceptions and judgments—they term this, “data saturation”—provided the respondents share some degree of expertise in the “domain of inquiry”.^17^ 12 respondents in this purposive sample may be considered high to moderately competent in this domain based on their occupations—ranging from medical doctor to community-based environmental scientist to directors of environmental non-profits—reinforcing our estimate that facts and inferences drawn from these interviews are essentially accurate.
- We maintained a consistent interview structure (same questions, same order) through out the process. Direct experience with the content of our interview questions was widely distributed throughout our pool of respondents. Our respondent pool was homogenous, insofar as all interviewees lived in severely impacted areas and worked actively in community/personal recovery efforts.
- There were some variances in respondent credibility/reliability. This correlated closely with respondent levels of engagement with community-based environmental issues prior to the disaster.
- While “lay expert” respondents represented a wide spectrum of the Louisiana environmental community—Southern Mutual Self-Help Association, Louisiana Environmental Action Network, New Orleans National Bucket Brigade Coalition, MacArthur and Heinz award-winning community-based environmental scientists, and Principal Chief of the United Houma Nation—it is always problematic to accept the opinions and agenda of any individual or organization as the surrogate “voice” of a community.
- Respondent interviews showed variability in environmental health knowledge and personal concern baselines. Respondents from our original purposive sample^12^ articulated crosscutting connections among toxic exposure and health effect linkages, social issues and damage to coast, estuaries and marshlands. More informally selected respondents^3^ usually showed more singular focus on personal food/shelter issues: reentry timelines, debris removal, rehabilitating flood damaged homes, and a quick return to pre-Katrina/Rita structure in their lives.

## Results

Survey responses fell into three general categories of information: general conclusions, environmental health risk perceptions/risk communication gaps, and possible formats for community research and intervention projects. Categories overlap and responses to the same survey item yielded information that could often be used verbatim, or as the basis for inferences and generalizations across categories.

Survey items #1, #2 and #3 were most useful in eliciting these broad stroke characterizations: 1) Interviews and commentary showed a wide variation in risk perceptions and degree of acceptable risk, 2) within this basically nonscientist population, the degree of risk anxiety and skepticism toward official risk assessments appears directly correlated with level of environmental health awareness and scientific literacy, 3) risk perceptions continue to evolve as monitoring yields credible data and, conversely, 4) credibility issues influence risk perception and mediate risk communication. (see Table 2)

While most of these findings are fairly straightforward, 2) requires further explanation of the seeming relationships among scientific backgrounds of individual respondents, their perceptions of risk, and their willingness to accept official risk assessments. Respondents with a comprehensive overview of the scope of storm damage, a working knowledge of National Priority List sites in the impacted areas, and a grasp of the extent of multi-media pollution in the petrochemical belt of coastal Louisiana and Mississippi prior to Hurricanes Katrina and Rita were most concerned that accurate risk assessments and citizen safety were lower priorities for local and state governments than jump-starting a severely damaged regional economy.

These beliefs were reinforced for respondents participating in community-based toxicity monitoring projects, even when community results roughly approximated data generated by Louisiana Department of Environmental Quality and EPA. One survey respondent, also a renowned environmental scientist managing community level monitoring projects in Louisiana and Mississippi, corroborated much of the agency data with her own results but diverged from official interpretations of how much risk these data implied, saying:

“… if it was a Superfund site, and the concentrations were at the levels we’re finding, they wouldn’t allow people to go back in there. They would require that the material be removed, treated and detoxified”.^18^

Other respondents with environmental health backgrounds independently echoed this sentiment and stressed the need for continuous and comprehensive monitoring, and a careful program of reentry and redevelopment. Conversely, respondents with less science background or community involvement were more focused on the practicalities of debris removal and rebuilding, and the speed and smoothness of the recovery.

Responses to items #2 and #3 provided most of our specific data on perceived health risks, gaps, ambiguities or inconsistencies in interpretation of data or formal communication of environmental health risks. Respondents covered a wide spectrum of possible toxic point sources, exposure pathways, and opinions on linkages with short and long term health effects. Many responses were in some sense linked to local geography and economics but *damage to coastal marshlands, mold exposure, respiratory effects of desiccated storm sludge, possible transport of metal residues and petrochemical waste, water quality and short-term/chronic mental health issues* were mentioned by 13 of 15 respondents.^19^

Responses to item #4 addressed the possibilities for collaborative responses to immediate damage, health and environmental monitoring, and preparation for the future. This query prompted a variety of elaborations including: *environmental health-oriented symptoms or biomarker surveys, monitoring toxic residue levels, and comprehensive hazard assessment and preparation.* Respondents who favored monitoring of toxicity and health effects expressed a strong preference for direct community involvement in all aspects of these processes, including formal risk assessments, and transparent, timely access to all results.^20^

Item #5 was incorporated into interviews with eight individuals involved with environmental and other community-based organizations including: Two Executive Directors of community-based environmental organizations, one Tribal Executive, one former Biology Professor/environmentalist at Southern University, Baton Rouge, one group practice community M.D., one private sector environmental scientist, one retired community/state Attorney General’s liaison specialist, one director of a private community health research non-profit. *All agreed that their missions and energies were redirected from long-term environmental projects to the immediate rescue/recovery response, and would be for the foreseeable future.*

## Outcomes and Applications

1) Based on assessments of environmental risk and community needs identified in responses to items #1, #2 and #3, the Sealy Center for Environmental Health and Medicine at UTMB agreed to assist the Louisiana Environmental Action Network with funding for production of additional reentry safety kits for citizens returning to homes inside the storm impact zones. The LEAN kits consist of disposable N-95 mask/respirators, safety goggles, disposable nitrile gloves, heavy-duty work gloves, a Tyvek suit and booties, and Clorox ultra. This kit was “co-authored” by Wilma Subra (Southern Mutual Help Association, Subra Company, Inc; New Iberia LA) and Mary Lee Orr (Executive Director: Louisiana Environmental Action Network; Baton Rouge LA) and thousands of kits have been distributed to returning home owners and volunteers who traveled to Louisiana from other regions to assist in recovery efforts.

2) Relevant responses to survey item #4 were analyzed, and compiled as a community “wish list” of necessary and urgent collaborative environmental health research projects. This compilation was presented in a special Katrina outreach panel convened at the NIEHS Center Director’s meeting (Oct. 29–Nov. 1; Vanderbilt University, Nashville TN). (see Table 3)

The panel consisted of representatives from NIEHS teams monitoring water borne pathogens, metals and petrochemical pollutants, and mold spores, as well as the director of the NIEHS Center Community Outreach and Education Core at UTMB who supervised outreach efforts, on location.^15^ Community representatives were not included on this panel as the need for rapid response stymied efforts to organize a more inclusive presentation. The 2006 NIEHS Environmental Justice/Community Based Participatory Research Grantees Meeting (Oct. 2006; Research Triangle Park, NC) featured a special panel consisting of Bishop James Black (Center for Environmental and Economic Justice, Biloxi MS), Wilma Subra, MS (Subra Company; New Iberia LA) and Paul Renner (The Labor Institute, collaborator with United Steel Workers and the Deep South Center for Environmental Justice on the “Safe Way Back Home” project).

3) UTMB-NIEHS pilot funding was approved to produce **Project CEHRO—Community Environmental Health and Risk Outreach**. This outreach was inspired by a survey recommendation by Willie Fontenot (Baton Rouge) that called for “*development of a disaster management plan and precautionary procedures that incorporate environmental health, risk communication and community hazards assessment.*”

Project CEHRO sparked collaboration among various local and regional agencies including: Les Reflections du Bayou, the United Houma Nation, Terrebonne-Lafourche Levee Districts, South Lafourche Unified School District, Bayou Inter-Faith Shared Community Organizing, Bayou Grace, Louisiana Spirit, Inner-Works, Inc. and more. The project offered 18 hours of training to a cadre of community volunteers using materials and techniques drawn from the ATSDR Toxicology Curriculum for Communities, the UTMB/NIEHS Center “Tox and Risk” Community Environmental Forum Project, the EPA NEJAC Cumulative Risk/Multiple Stressor Interaction Matrix, and the Louisiana Department of Health and Hospitals. UTMB/NIEHS community outreach personnel created a site-specific handbook for the training that incorporated themes and topics developed consensually by community project partners; major themes included:

- Wetlands Loss and Hurricane Evacuation Safety
- Mental Health Issues
  – Disasters and Children: a Developmental Approach
  – Handling Disaster News
  – Post-Traumatic Stress and Disaster Anxiety
  – Vulnerable Groups
- Toxic Exposures and Medical Issues: an Epidemiology of Hurricanes
- Assessing Community Hazards, Understanding Risk Perceptions, Communicating Risk

This instructional program, geared toward site-specific treatment of a range of consequences stemming from severe storms, prepared volunteers to serve as peer educators and risk communicators in their respective communities.^21^

UTMB-NIEHS Public Forum and Toxics Assistance Division conducted the *Tox and Risk* segment of the workshop; local mental health and group leadership practitioners co-facilitated sections on Community Development and Advocacy/Risk Communication. South Lafourche Levee District and EPA personnel covered effects of previous flood management and channel engineering interventions on coastal marshland, and gave an overview of coastal reclamation plans promoted by various governmental agencies and nonprofits. Private sector and university environmental health scientists clarified results of soil and water monitoring, CDC tracking of epidemiological trends, and community hazard assessments. This curriculum developed through a consultative process involving major project partners: UTMB NIEHS Center, South Lafourche School District, United Houma Nation, Bayou Interfaith Shared Community Organizing.^16^,^18^–^20^ Workshops were presented in Gray, Houma, Thibodaux, and Chauvin (Terrebonne Parish), and Galliano (Lafourche Parish).

## Conclusions

Risk uncertainty, ambiguities in risk assessment and lack of access to reliable information on risks and precautions contribute to risk anxiety and complicate regional, local and individual reentry strategies. Complications in mapping storm surge exposure pathways compound this dilemma.^9^,^10^,^14^,^16^ Florence Robinson (Baton Rouge), winner of a Heinz Award in the Environment, underscored the effects of dislocation, hyper-vigilance, fear of the future, and of “leading permanently temporary lives” in undermining the mental health of evacuees, especially children. Mary Lee Orr noted the pressure of social stressors in producing “disaster fatigue” and contributing to “increased mental illness, suicide, drinking and drug abuse.” The lack of safe housing, a possibly biased allocation plan for recovery resources, and the fate of communities beyond the pale of levee systems were mentioned by respondents from New Orleans to Dos Gris.

The preexisting “trust dynamic” among citizens and regulatory entities charged with protecting population health and the environment certainly mediates the reception of official risk communication.^20^–^22^ In general, environmental justice veterans and members of fence-line communities carrying heavy pre-Katrina burdens of cumulative risk regarded health and safety pronouncements from the Agency for Toxic Substances and Disease Registry, the Environmental Protection Agency, the Louisiana Department of Environmental Quality, and private industry with deep skepticism based on disappointments, perceived betrayals and bitter experience with prior institutional versions of the truth.^22^,^23^ Anne Rolfes (New Orleans/Chalmette), Program Director of the Louisiana Bucket Brigade, captured this skepticism, observing “people are still in shock, they’re in a state of suspended unknowing because they don’t know what’s true, they don’t know where to turn.” MacArthur Award-winning analytic chemist, Wilma Subra (New Iberia), countered LDEQ admonitions that, in general, post-Katrina toxic levels were essentially similar to what obtained before the storm with her own informed prescription:

“… sediment sludge carried onto the land by the storm surge is contaminated by heavy metals and a host of microorganisms, all of which are known to cause acute and chronic impacts on public health. There is a need to determine the extent of that contamination and establish a plan to remove the contaminants in order to prevent residents and workers from being harmfully exposed.”^24^

Contention over issues such as the storm surge enabling effect of the Mississippi River Gulf Outlet (MR. GO), the significance of Murphy Oil spill data for citizens of Chalmette LA^22^,^23^,^25^ and the use of decommissioned landfills (*Gentilly*) or landfills closely proximate to minority neighborhoods (*Chef Menteur*) was heated and sharp.^26^ In the eyes of many environmentally engaged citizens, these issues remain unresolved.

In some degree, all respondents were acutely aware that levee systems, industrial channels, and the Mississippi River diversion adversely affect the capacity of native marshland to dampen hurricane force winds and absorb storm surge? This was a serious issue throughout all coastal areas: Plaquemines, Jefferson, St. Bernard, and Orleans parishes (Katrina), and Cameron, Vermillion, Terrebonne and Lafourche parishes LA, as well as Jefferson, Orange and Chambers counties in east Texas (Rita).^19^,^27^,^28^ The Louisiana Coastal Area Feasibility Study seeks to address landform restoration, infrastructure protection, and water movement modeling to avoid a reprise of 2005.^27^–^29^ But time is short, and the possible effects of climate change on Gulf storm patterns complicate available models and cloud our collective window into the future of our Third Coast.

## Figures and Tables

**Table 1.** Ecological/social damage assessment, risk perceptions, environmental collaborations interview items.

What significant damage did your area sustain during or because of Hurricane Katrina and/or Rita? What are the most significant threats to human health in your area, post-Katrina (or Rita)? How has the hurricane evacuation, reentry and recovery process disrupted the social fabric of your area, and Louisiana, generally? What environmental health projects—involving collaborations among environmental scientists, health care and social service providers, and communities—do you think are most important to safeguard the health of people and the environment in your area, your region, your state? Describe your organization’s response to this disaster. How have you modified your mission to make a effective response? How have these modifications affected your org’s capacity to realize your original mission? (Applicable only in interviews with directors/members of environmental orgs.)

**Table 2.** Perceived short term/long term environmental health risks.

exposure to mold: concentrations, direct exposure effects, possible immuno-suppression and recommended precautions massive loss of marsh and wetlands, loss of marshland’s hurricane dampening effect water potability during and immediately post-disaster levels of heavy metals, diesel and hydrocarbon residues in desiccated sludge; change in levels over time respiratory and other health effects of wind-borne sludge dust (*Katrina cough*) transport of petrochemical toxicants and metal residues moved by storm surges effects of damage to coastal marsh on subsistence food supply and health of the estuarine eco-system depression, disorientation, post-traumatic stress effects of disaster effects of disaster stressors on the most vulnerable segments of the population: children, the elderly, disabled individuals post-Katrina viability of drinking water treatment facilities extent of threat from pathogens in water; ongoing monitoring of pathogen levels in bayou surface water and major bodies such as Lake Pontchartrain; rashes and lesions as consequence of immersion in flood water dispersion patterns and health effects of toxic releases from submerged automobiles, agricultural chemicals, non-petrochemical industrial sites viability and potential virulence of pathogens in desiccated sludge integrity of superfund sites and brown fields after wind damage and submergence; effects on prior capping or other abatement remedies flooding/overflow risk to surface water from RCRA-exempt waste pits and compromised sewage treatment facilities post wind and storm-surge integrity of petrochemical facilities; direct damage to physical plants emissions and flaring during post-storm petrochemical shutdown-restart process; regulatory waivers on un-permitted emissions during start-up process debris issues: collection, transportation, certified and marginal disposal sites, use of air curtain destructors to contain emissions from incineration process need for specific reentry safety gear not clearly indicated; lack of information on reentry procedures; safety equipment unavailable; price gouging mosquitoes, animal and/or human corpses, diseased animals as contagious disease vectors location of “permanently temporary” FEMA-villes proximate to point sources of air toxics or waste disposal areas permanent reconfiguration of regional political demographic with consequent effects on redevelopment policy and environmental justice issues

**Table 3.** Katrina/rita community research collaboration environmental health and safety “wish list”.

Monitoring health outcomes using combination biomarker assay and health effects survey with rescue and recovery workers. Differentiation of acute/chronic health effects in recovery workers based on time period of response, length of time working in impact area, proximity to documented environmental impact areas, occupational category etc. This monitoring could also be extended to ALL residents re-entering affected areas. Systematic bio monitoring of vulnerable populations returning to high impact areas. Results used to develop individual clinical intervention plans where applicable and track overall population health. Development of a comprehensive—but simultaneously site-specific—disaster management plan and procedures that incorporate statewide environmental risk communication, and hazard preparation training for community-based environmental organizations. Monitoring infiltration of potable water supplies (with emphasis on bayou supplied communities) by petrochemical releases, effluents and waste disposal site residues. Monitoring equipment would remain on-site, on-line with opportunities for continuous data feed and future emergency response measurements. Multi-agency efforts to create an inter-coastal and marshland reclamation waterway policy that sustains industrial economy while preventing further salt water infiltration of marshlands and restores essential storm buffering. Public Forums on waste disposal efforts to address hazardous and non-hazardous debris removal, storage/sequestration, and/or incineration. Occupational risk survey of Latino workers (documented and undocumented) to establish pre-exposure baselines (as possible), exposure pathways and levels of exposure as consequences of recovery employment.

**Table 4.** “Public talks science listens…” project timeline.

October 8th–25th (2005)	UTMB NIEHS COEC/MD Anderson-UT/Smithville CRED(2005) deploys outreach teams in South Louisiana
October 31st–November 1st (2005)	Presentation of interview results at NIEHS Center Directors Meeting by Pam Diamond, UTMB/NIEHS COEC Director; VanderBilt University; Nashville TN
April 14 (2006)	Hurricane Readiness Pilot Project funded through National Institute for Environmental Health Sciences Center in Environmental Toxicology@UTMB (ES006676)
May–August (2006)	Curriculum/Logistical planning with community partners in Terrebonne/Lafourche Parishes, Louisiana
September 8th–12th (2006)	Rollout of Project CEHRO (Community Environmental Health and Risk Outreach) in Gray, Houma (Terrebonne), and Galliano (Lafourche)
March 17th–18th (2007)	Supplemental workshop reflecting evolution of EPA risk assessment and recovery facts on the ground, Chauvin (Terrebonne)
March 31st (2007)	End of project funding period

## Appendix of Tables.

**Table 1.** Risk assessment-perceptions, environmental collaborations survey.

What significant damage did your area sustain during or because of Hurricane Katrina and/or Rita? What is the most significant threat to human health in your area, post-Katrina (or Rita)? How has the hurricane evacuation, reentry and recovery process disrupted the social fabric of your area, and Louisiana, generally? What environmental health projects—involving collaborations among environmental scientists, health care and social service providers, and communities—do you think are most important to safeguard the health of people and the environment in your area, your region, your state? Describe your organization’s response to this disaster. How have you modified your mission to make a effective response? How have these modifications affected your org’s capacity to realize your original mission? (Applicable only in interviews with members of environmental orgs.)

**Table 2.** Perceived short term/long term environmental health risks.

exposure to mold: concentrations, direct exposure effects, possible immuno-suppression and recommended precautions massive loss of marsh and wetlands, loss of marshland’s hurricane dampening effect water potability during and immediately post-disaster levels of heavy metals, diesel and hydrocarbon residues in desiccated sludge; change in levels over time respiratory and other health effects of wind-borne sludge dust (*Katrina cough*) transport of petrochemical toxicants and metal residues moved by storm surges effects of damage to coastal marsh on subsistence food supply and health of the estuarine eco-system depression, disorientation, post-traumatic stress effects of disaster effects of disaster stressors on the most vulnerable segments of the population: children, the elderly, disabled individuals post-Katrina viability of drinking water treatment facilities extent of threat from pathogens in water; ongoing monitoring of pathogen levels in bayou surface water and major bodies such as Lake Pontchartrain; rashes and lesions as consequence of immersion in flood water dispersion patterns and health effects of toxic releases from submerged automobiles, agricultural chemicals, non-petrochemical industrial sites viability and potential virulence of pathogens in desiccated sludge integrity of superfund sites and brown fields after wind damage and submergence; effects on prior capping or other abatement remedies flooding/overflow risk to surface water from RCRA-exempt waste pits and compromised sewage treatment facilities post wind and storm-surge integrity of petrochemical facilities; direct damage to physical plants emissions and flaring during post-storm petrochemical shutdown-restart process; regulatory waivers on un-permitted emissions during start-up process debris issues: collection, transportation, certified and marginal disposal sites, use of air curtain destructors to contain emissions from incineration process need for specific reentry safety gear not clearly indicated; lack of information on reentry procedures; safety equipment unavailable; price gouging mosquitoes, animal and/or human corpses, diseased animals as contagious disease vectors location of “permanently temporary” FEMA-villes proximate to point sources of air toxics or waste disposal areas permanent reconfiguration of regional political demographic with consequent effects on redevelopment policy and environmental justice issues

**Table 3.** Katrina/Rita community research “Wish List”.

Monitoring health outcomes using combination biomarker assay and health effects survey with rescue and recovery workers. Differentiation of acute/chronic health effects in recovery workers based on time period of response, length of time working in impact area, proximity to documented environmental impact areas, occupational category etc. This monitoring could also be extended to ALL residents re-entering affected areas. Systematic bio monitoring of vulnerable populations returning to high impact areas. Results used to develop individual clinical intervention plans where applicable and track overall population health. Development of a comprehensive—but simultaneously site-specific—disaster management plan and procedures that incorporate statewide environmental risk communication, and hazard preparation training for community-based environmental organizations. Monitoring infiltration of potable water supplies (with emphasis on bayou supplied communities) by petrochemical releases, effluents and waste disposal site residues. Monitoring equipment would remain on-site, on-line with opportunities for continuous data feed and future emergency response measurements. Multi-agency efforts to create an inter-coastal and marshland reclamation waterway policy that sustains industrial economy while preventing further salt water infiltration of marshlands and restores essential storm buffering. Public Forums on waste disposal efforts to address hazardous and non-hazardous debris removal, storage/sequestration, and/or incineration. Occupational risk survey of Latino workers (documented and undocumented) to establish pre-exposure baselines (as possible), exposure pathways and levels of exposure as consequences of recovery employment.

**Table 4.** “Public talks science listens…” project timeline.

October 8th–25th (2005)	UTMB NIEHS COEC/MD Anderson-UT/Smithville CRED(2005) deploys outreach teams in South Louisiana
October 31st–November 1st (2005)	Presentation of interview results at NIEHS Center Directors Meeting by Pam Diamond, UTMB/NIEHS COEC Director; VanderBilt University; Nashville TN
April 14 (2006)	Hurricane Readiness Pilot Project funded through National Institute for Environmental Health Sciences Center in Environmental Toxicology@UTMB (ES006676)
May–August (2006)	Curriculum/Logistical planning with community partners in Terrebonne/Lafourche Parishes, Louisiana
September 8th–12th (2006)	Rollout of Project CEHRO (Community Environmental Health and Risk Outreach) in Gray, Houma (Terrebonne), and Galliano (Lafourche)
March 17th–18th (2007)	Supplemental workshop reflecting evolution of EPA risk assessment and recovery facts on the ground, Chauvin (Terrebonne)
March 31st (2007)	End of project funding period
